# Epidemiologic Shift in Candidemia Driven by *Candida auris*, South Africa, 2016–2017^1^

**DOI:** 10.3201/eid2509.190040

**Published:** 2019-09

**Authors:** Erika van Schalkwyk, Ruth S. Mpembe, Juno Thomas, Liliwe Shuping, Husna Ismail, Warren Lowman, Alan S. Karstaedt, Vindana Chibabhai, Jeannette Wadula, Theunis Avenant, Angeliki Messina, Chetna N. Govind, Krishnee Moodley, Halima Dawood, Praksha Ramjathan, Nelesh P. Govender, for GERMS-SA

**Affiliations:** National Institute for Communicable Diseases, Johannesburg, South Africa (E. van Schalkwyk, R.S. Mpembe, J. Thomas, L. Shuping, H. Ismail, N.P. Govender);; Vermaak & Partners–Pathcare Pathologists, Johannesburg (W. Lowman);; Wits Donald Gordon Medical Centre, Johannesburg (W. Lowman);; University of the Witwatersrand, Johannesburg (W. Lowman, A.S. Karstaedt, V. Chibabhai, J. Wadula, A. Messina, N.P. Govender);; University of Pretoria and Kalafong Provincial Tertiary Hospital, Pretoria, South Africa (T. Avenant); Netcare Hospitals Ltd, Johannesburg (A. Messina);; Lancet Laboratories, Durban, South Africa (C.N. Govind, K. Moodley);; University of KwaZulu-Natal, Durban (C.N. Govind, H. Dawood, P. Ramjathan);; Grey’s Hospital, Pietermaritzburg, South Africa (H. Dawood);; National Health Laboratory Service, Johannesburg (V. Chibabhai, J. Wadula, P. Ramjathan)

**Keywords:** Candida, Candida auris, candidemia, mycoses, South Africa, fungi, multidrug resistance, antifungal drug resistance, antimicrobial resistance

## Abstract

*Candida auris* is an invasive healthcare-associated fungal pathogen. Cases of candidemia, defined as illness in patients with *Candida* cultured from blood, were detected through national laboratory-based surveillance in South Africa during 2016–2017. We identified viable isolates by using mass spectrometry and sequencing. Among 6,669 cases (5,876 with species identification) from 269 hospitals, 794 (14%) were caused by *C. auris*. The incidence risk for all candidemia at 133 hospitals was 83.8 (95% CI 81.2–86.4) cases/100,000 admissions. Prior systemic antifungal drug therapy was associated with a 40% increased adjusted odds of *C. auris* fungemia compared with bloodstream infection caused by other *Candida* species (adjusted odds ratio 1.4 [95% CI 0.8–2.3]). The crude in-hospital case-fatality ratio did not differ between *Candida* species and was 45% for *C. auris* candidemia, compared with 43% for non–*C. auris* candidemia. *C. auris* has caused a major epidemiologic shift in candidemia in South Africa.

Since 2009, when the first case of *Candida auris* infection was identified in South Africa, the number of laboratory-confirmed cases has increased exponentially (*1*). This multidrug-resistant fungal pathogen emerged worldwide, appearing almost simultaneously on 6 continents, causing invasive disease and protracted healthcare-associated outbreaks (*2*–*5*). The reported crude case-fatality ratio among patients with invasive *C. auris* infections is high, although the attributable mortality rate has not been determined (*3*,*6*). *C. auris* persists on surfaces, is transmitted among patients in the healthcare environment, forms biofilms, and resists routinely used environmental cleaning agents (*7*–*10*). *Candida* spp. are a common cause of bloodstream infections and were responsible for 13% (95% CI 6%–26%) of healthcare-associated bloodstream infections according to a 2015 US point-prevalence survey (*11*). *C. parapsilosis* was the dominant species causing candidemia according to a national survey in South Africa conducted during 2009–2010 (*12*). Patients at risk for candidemia in general are the critically ill (especially premature neonates) and those with serious underlying illnesses (e.g., diabetes mellitus and hematologic malignancies), prior or prolonged exposure to broad-spectrum antimicrobial drugs, and invasive medical and surgical interventions (*13*). Previously described characteristics associated with candidemia among adults in South Africa included abdominal surgery, trauma, diabetes mellitus, cancer, and HIV infection (*14*). *C. auris* is thought to occupy a similar niche in the healthcare environment as *C. parapsilosis* because both organisms colonize human skin and adhere to healthcare surfaces and devices. Clinical risk factors for *C. auris* infection would be expected to be similar to those for *C. parapsilosis* infection, but these factors are largely reported from several small case series. Risk factors for *C. auris* candidemia (compared with other species) among patients admitted to 27 intensive care units in India included underlying respiratory disease, vascular surgery, having a urinary catheter in situ, prior antifungal drug exposure, and a low APACHE II score at admission (*6*). In South Africa, most reported cases of *C. auris* colonization or invasive disease occurred in older patients (median age 60 years) (*1*) (R.E. Magobo, National Institute for Communicable Diseases [NICD], South Africa, pers. comm., 2019 Jul 1). To inform infection prevention and empiric antifungal treatment strategies, we used national surveillance data for South Africa to estimate the total incidence risk for candidemia and the proportion of candidemia cases caused by *C. auris* and to determine factors associated with *C. auris* candidemia compared with other *Candida* species,

## Materials and Methods

### Surveillance for Candidemia

From January 1, 2016, through December 31, 2017, we conducted active national laboratory-based surveillance for candidemia by using the NICD GERMS-SA surveillance platform. We requested that *Candida* species from any episode of bloodstream infection, with an accompanying laboratory report (including basic patient demographic data), be submitted from all clinical microbiology laboratories within the National Health Laboratory Service (NHLS), a national public-sector laboratory network, and from all pathology laboratory practices in the private sector. We have previously described the methods used by private and NHLS laboratories for species identification (*1*). Isolates were sent to the NICD’s Mycology Reference Laboratory for confirmation of identification and antifungal drug susceptibility testing. In addition, surveillance officers (nurses or pharmacists) collected basic clinical and demographic data on standardized electronic case report forms at 22 public-sector and 3 private-sector enhanced surveillance sites, all of which were large acute-care hospitals. We did not collect sufficient data to define severity of illness scores (e.g., APACHE II or McCabe scores). We conducted retrospective audits to ensure complete case ascertainment.

We extracted line list data from the laboratory information systems of NHLS and private laboratories, compared those data with reported cases, deduplicated the data (by using patient name, surname, date of birth, hospital number, and specimen collection date), and added missing cases to the surveillance database. For cases detected by audit, we recorded the *Candida* species identification reported by the reporting laboratory. In 2013, the estimated number of beds in private-sector hospitals nationwide was 34,572, of which 45% were located in Gauteng Province, the most economically active and densely populated province of South Africa (*15*).

### Case Definitions

We defined a case of candidemia as illness in any patient at a healthcare facility in South Africa who had *Candida* species isolated from a blood culture specimen processed by an NHLS or private-sector diagnostic laboratory. We defined a confirmed case of *C. auris* candidemia as illness in a patient with an isolate confirmed as *C. auris* at NICD, regardless of the referring laboratory’s initial identification. We also included probable cases for which the referring laboratory identified *C. auris* or *Candida haemulonii* but a viable isolate was not available for confirmation at NICD. Multiple *Candida* isolates cultured within 30 days of the first positive blood culture specimen were included in a single case. We classified cases of candidemia into 2 groups on the basis of NICD identification (or the referring laboratory’s identification if a viable isolate was not available): *C. auris* and non–*C. auris* candidemia.

### Reference Laboratory Methods

Isolates were submitted to NICD on Dorset transport medium (Diagnostic Media Products, http://www.nhls.ac.za). For viable isolates, species-level identification was confirmed by using matrix-assisted laser desorption/ionization time-of-flight (MALDI-TOF) mass spectrometry (Bruker Corporation, https://www.bruker.com). We amplified and sequenced the internal transcribed spacer or D1/D2 region of the ribosomal gene for isolates when MALDI-TOF mass spectrometry did not yield a score >2.

### Statistical Analyses

We calculated the overall incidence risk for candidemia for hospitals for which admissions data were available, stratified by healthcare sector, by dividing the total number of new cases of candidemia by the total number of hospital admissions (i.e., number of persons at risk) in each sector for the 2-year period. We also calculated healthcare facility incidence risk per hospital when admission denominator data were available. We obtained admissions data by directly approaching private hospital groups and through the GERMS-SA surveillance platform for public-sector hospitals. We used ArcGIS mapping software (https://www.esri.com) to plot the location and number of *C. auris* candidemia cases at hospitals in Gauteng Province and used inverse distance-weighted interpolation to map hotspot hospitals, which we defined as those with >10 reported cases of *C. auris* candidemia during the 2-year period.

We hypothesized a priori that systemic azole exposure was associated with candidemia caused by *C. auris* rather than other *Candida* species. Distinguishing cases of *C. auris* candidemia from those caused by other species is important to physicians choosing an empiric antifungal treatment regimen for suspected candidemia and to infection prevention and control practitioners for rapid identification of cases requiring contact precautions. We used multivariable logistic regression to assess this association among patients admitted to 25 enhanced surveillance sites.

We compared proportions between groups by using a χ^2^ or Fisher exact test. We compared medians by using a Wilcoxon rank-sum test.

### Ethics

NICD obtained annual approval for GERMS-SA laboratory-based surveillance from the human research ethics committees of several universities in South Africa. Patients from whom surveillance data were collected prospectively through interview provided written informed consent.

## Results

**Figure 1:**
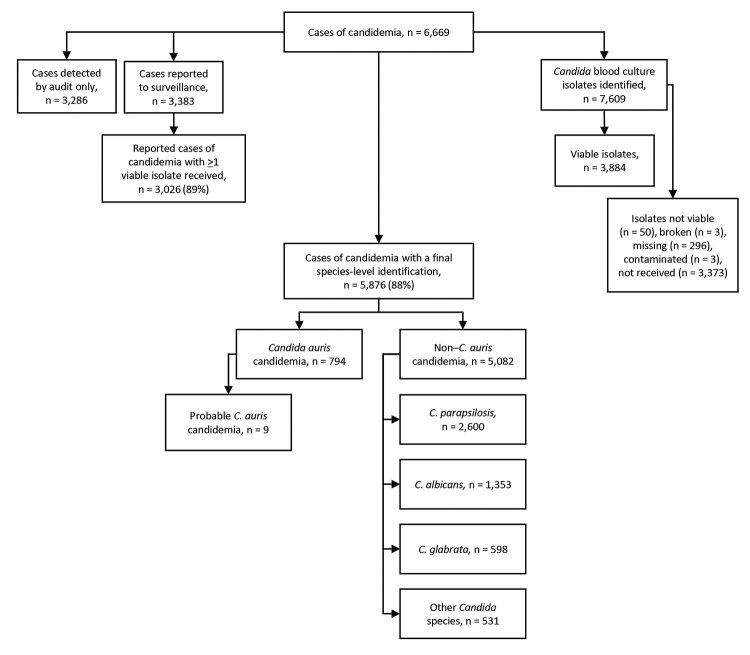
Flowchart showing numbers of candidemia cases detected by national surveillance and *Candida* species identified, South Africa, 2016–2017.

During the 2-year surveillance period, 6,669 cases of candidemia (6,629 first and 40 recurrent episodes) were detected across South Africa at 103 public-sector and 166 private-sector hospitals (2,529 cases [38%] in the public sector, 4,140 cases [62%] in the private sector). Of the 6,669 cases, viable isolates were identified to species level at NICD for 3,020 (45%) cases. Species identification was available for a further 2,856 cases (2,842 from private laboratories, 14 from NHLS laboratories). Among 5,876 cases with a species-level identification, 794 (14%) were caused by *C. auris* and 5,082 (86%) by other *Candida* species (Figure 1). The most common *Candida* species in the non–*C. auris* group were *C. parapsilosis* (2,600 [44%]), *C. albicans* (1,353 [23%]), *C. glabrata* (598 [10%]), *C. tropicalis* (140 [2%]), and *C. krusei* (98 [2%]). Twenty-nine cases had a mixed episode of candidemia caused by *C. auris* and another *Candida* species (mostly *C. parapsilosis* [21 cases]).

The total incidence risk for candidemia (expressed as cases/100,000 hospital admissions) at 115 private-sector and 18 public-sector hospitals with available admissions data was 71.2 (95% CI 68.6–73.8) in the private sector and 149.5 (95% CI 141.1–158.1) in the public sector, for a total of 83.8 (95% CI 81.2–86.4]) (Table 1). Incidence risk for *C. auris* was 13.6 (95% CI 12.4–14.8) in the private sector, compared with 6.9 (95% CI 5.2–9.0) in the public sector; incidence risk ratio was 1.96 (95% CI 1.4–2.6). Individual healthcare facility incidence risk ranged from 2.6 to 375 for *C. parapsilosis*, 1.3 to 221 for *C. albicans*, 0.9 to 154 for *C. auris*, and 1.7 to 107 for *C. glabrata*.

**Table 1:** Incidence risk for candidemia at a limited number of public- and private-sector hospitals with available admissions data, by *Candida* species and healthcare sector, South Africa, 2016–2017*

*Candida* species	**No. cases at 133 hospitals**	**Total incidence risk† (95% CI)**	**Incidence risk at 18 public-sector hospitals† (95% CI)**	**Incidence risk at 115 private-sector hospitals† (95% CI)**	**Incidence risk ratio, private sector:public sector (95% CI)**
*C. parapsilosis*	1,657	32.98 (31.3–34.6)	27.98 (24.4–31.9)	33.94 (32.2–35.8)	1.21 (1.0–1.4)
*C. albicans*	735	14.63 (13.5–15.8)	34.55 (30.6–38.9)	10.82 (98.5–11.9)	0.31 (0.2–0.4)
*C. auris*	628	12.50 (11.5–13.6)	6.93 (5.2–9.0)	13.57 (12.4–14.8)	1.96 (1.4–2.6)
*C. glabrata*	352	7.01 (6.2–7.8)	12.13 (9.8–14.8)	6.02 (5.31–6.9)	0.50 (0.3–0.7)
Other	308	6.13 (5.4–6.9)	13.25 (10.8–16.1)	4.77 (4.1–5.5)	0.36 (0.2–0.5)
Total‡	4,209	83.78 (81.2–86.4)	149.46 (141.1–158.1)	71.20 (68.6–73.8)	0.48 (0.4–0.6)

*Admissions data were available for 115 private-sector hospitals (4,216,306 admissions) and 18 public-sector hospitals (807,600 admissions). †No. cases/100,000 hospital admissions. ‡A total of 529 candidemia cases had no *Candida* species identified, and incidence risk for these are not displayed in the table. However, these case numbers are included in the total number of candidemia cases and total incidence risk calculations.

We received 4,236 isolates from 70 NHLS laboratories and 4 amalgamated private-sector pathology practices, and we identified an additional 3,307 cases (with 3,373 corresponding isolates) by retrospective audits. Of the 400 confirmed viable *C. auris* isolates received, 258 (65%) had an initial identification of *C. auris*.

**Figure 2:**
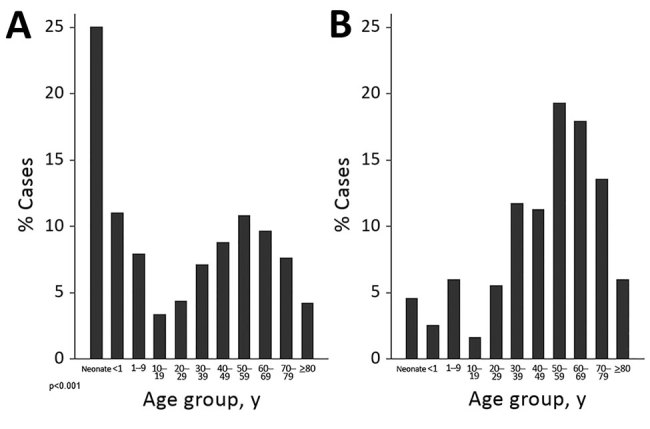
Age distribution of case-patients with candidemia caused by *Candida auris* compared with other *Candida* species, South Africa, 2016–2017. A) *C. auris* patient median age was 54 years (interquartile range 34–67 years); B) other *Candida* species patient median age was 27 years (interquartile range 0–57 years).

Among 435 patients with *C. auris* candidemia for whom data were available (including 9 patients with probable *C. auris* infection), the median age was 54 years (interquartile range [IQR] 34–67 years), compared with a median of 27 years (IQR 0–57 years) among 4,050 patients with non–*C. auris* candidemia (p<0.001) (Table 2; Figure 2). Neonates comprised the largest proportion of patients with non–*C. auris* candidemia (1,015/4,050; 25%), whereas only 20 cases (5%) in the *C. auris* group were in neonates (Table 2). Of patients with *C. auris*, 61% (284/463) were male; 54% (1,729/3,216) were male in the non–*C. auris* group.

**Table 2:** Demographic and clinical characteristics of 6,669 patients with candidemia caused by *Candida auris* compared with other *Candida* species, South Africa, 2016–2017*

**Characteristics**	**All candidemia**	***C. auris***	**Non–*C. auris***	***C. parapsilosis***	***C. albicans***	***C. glabrata***
No. case-patients	6,669	794	5,875	2,600	1,353	598
Systemic antifungal drug therapy <14 d before positive culture†	317/1,829 (17.3)	30/95 (31.6)	287/1,734 (16.6)	108/477 (22.6)	36/441 (8.2)	11/166 (6.6)
Azole	219/317 (69.1)	16/30 (53.3)	203/287 (70.7)	72/108 (66.7)	30/36 (83.3)	9/11 (81.8)
Polyene/amphotericin B	38/317 (12)	7/30 (23.3)	31/287 (10.8)	12/108 (11.1)	5/36 (13.9)	0/11 (0)
Echinocandin	79/317 (24.9)	13/30 (43.3)	66/287 (23)	27/108 (25)	2/36 (5.6)	2/11 (18.2)
Age, y, median (IQR)	32 (0–58)	54 (34–67)	27 (0–57)	24 (0–58)	24 (0–56)	54 (32–67)
Sex						
Men and boys	2,013/3,679 (54.7)	284/463 (61.3)	1,729/3,216 (53.8)	806/1474 (54.7)	533/978 (54.5)	232/444 (52.3)
Women and girls	1,666/3,679 (45.3)	179/463 (38.7)	1,487/3,216 (46.2)	668/1,474 (45.3)	445/978 (45.5)	212/444 (47.7)
Length of hospital stay, d median (IQR)	32 (16–54)	55 (32–81)	31 (15–52)	40 (25–59)	24 (12–43)	22 (9–41)
Length of stay until first positive blood culture, d, median (IQR)	13 (5–24)	28 (15–46)	12 (5–23)	16 (10–27)	10 (3–19)	6 (1–16)
Province						
Gauteng	4,229/6,669 (63.4)	680/794 (85.6)	3,549/5,875 (60.4)	1,651/2,600 (63.5)	736/1,353 (54.4)	323/598 (54)
Other	2,440/ 6,669 (36.6)	114/794 (14.4)	2,326/5,875 (39.6)	949/2,600 (36.5)	617/1,353 (45.6)	275/598 (46)
Healthcare sector						
Public	2,529/6,669 (37.9)	99/794 (12.5)	2,430/5,875 (41.4)	599/2,600 (23)	673/1,353 (49.7)	248/598 (41.5)
Private	4,140/6,669 (62.1)	695/794 (87.5)	3,445/5,875 (58.6)	2,001/2,600 (77)	680/1,353 (50.3)	350/598 (58.5)
Hospital admission in past 12 mo	1,428/1,967 (72.6)	77/104 (74)	1,351/1,863 (72.5)	378/529 (71.5)	341/486 (70.2)	126/174 (72.4)
Intensive care unit admission	1,579/2,167 (72.9)	110/125 (88)	1,469/2,042 (71.9)	502/606 (82.8)	377/539 (69.9)	133/190 (70)
Mechanical ventilation	611/1,818 (33.6)	44/91 (48.4)	567/1,727 (32.8)	175/476 (36.8)	129/440 (29.3)	57/165 (34.6)
Central venous catheter in situ	1,031/1,817 (56.7)	69/92 (75)	962/1,725 (55.8)	289/479 (60.3)	229/443 (51.7)	89/165 (53.9)
Systemic antimicrobial drug therapy in 14 d before positive culture	1,292/1,830 (70.6)	77/94 (81.9)	1,215/1,736 (70)	349/481 (72.6)	284/441 (64.4)	105/164 (64.0)
Crude in-hospital case-fatality ratio	8,39/1,966 (42.7)	46/102 (45.1)	793/1,864 (42.5)	166/516 (32.2)	247/492 (50.2)	91/179 (50.8)

*Values are no. (%) except as indicated. The 3 most common *Candida* species in the non–*C. auris* group (*C. parapsilosis*, *C. albicans*, and *C. glabrata*) are shown separately for comparison. For the purpose of this analysis, cases of candidemia with no final species identification were included in the non–*C. auris* group. IQR, interquartile range. †Patients could have received >1 class of antifungal drug therapy.

**Figure 3:**
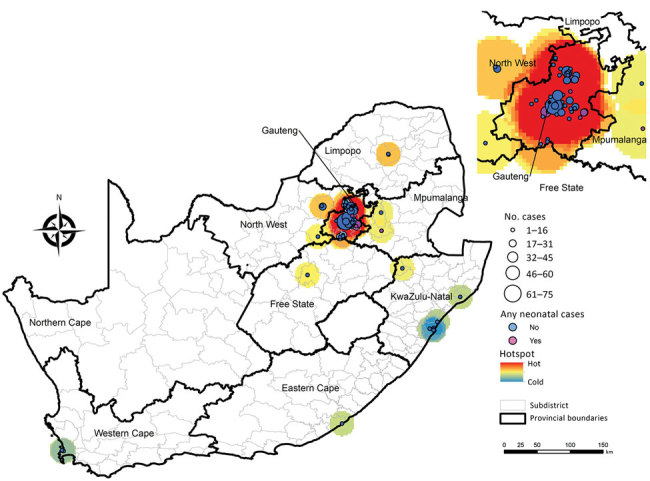
Location and number of 741 *Candida auris* candidemia cases at 79 hospitals, including 7 hospitals with neonatal cases, South Africa, 2016–2017. Location data were missing for 53 cases.

**Figure 4:**
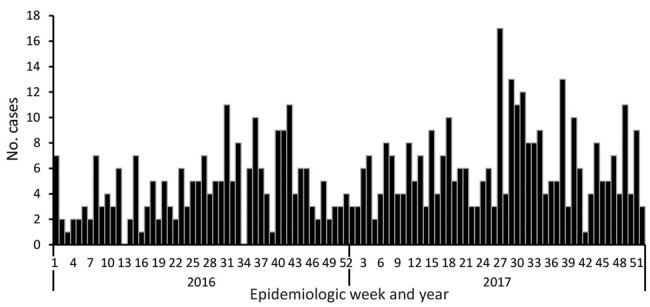
Cases of *Candida auris* candidemia (N = 557), by epidemiologic week, Gauteng Province, South Africa, 2016–2017. Date of blood culture collection was missing for 123 cases.

Most (86%, 680/794) cases of *C. auris* candidemia were from hospitals in Gauteng Province and 88% (695/794) from private-sector facilities, compared with 60% (3,549/5,875) in Gauteng Province and 59% (3,445/5,875) from private-sector facilities among non–*C. auris* cases. Cases of *C. auris* candidemia were diagnosed at 14 public-sector and 67 private-sector hospitals (Figure 3), most of which are located in Gauteng Province (Figure 4). Among these, 25 hospitals had >10 cases of *C. auris* candidemia during the 2-year period (meeting our definition of hotspot hospitals); the largest absolute number of cases was reported from a large private hospital and another large academic teaching hospital. However, incidence risk for *C. auris* candidemia was highest in a smaller private-sector hospital (13 cases/8,431 admissions [154 cases/100,000 admissions]). Of the 20 hospitals with the highest incidence, 19 were private-sector facilities. Several small outbreaks occurred at these hotspot hospitals, but in different wards within each hospital (data not shown).

We collected clinical data for 2,067 patients at enhanced surveillance sites, including 535 patients whose isolates were not identified at the species level. Most patients with *C. auris* bloodstream infections had received prior (<14 days before diagnosis) systemic antimicrobial drug therapy (77/94 [82%]), and 30/95 (32%) had received prior systemic antifungal drug therapy. Of the 30 patients with prior antifungal therapy, 16 had received azoles, 7 had received amphotericin B, and 13 had received echinocandins. Among 105 patients with *C. auris* candidemia for whom clinical data were available, the median length of hospitalization before onset of candidemia was 28 days (IQR 15–46 days), compared with 12 days (IQR 5–23 days) among 1,852 patients with non–*C. auris* candidemia (p<0.001). Approximately one third (32/105 [31%]) of patients with *C. auris* candidemia spent >6 weeks in hospital before the first positive blood culture was obtained. Seventy-seven (74%) patients with *C. auris* infection had been hospitalized in the past year, and 110 (88%) patients were admitted to an intensive care unit at some point during their current hospital stay. Eleven (26%) of 43 patients with *C. auris* candidemia were HIV-seropositive, similar to patients infected with other *Candida* species (251/972; 26%). The crude in-hospital case-fatality ratio did not differ between *Candida* species and was 45% for *C. auris* candidemia, compared with 43% for non–*C. auris* candidemia (p = 0.6) (*C. albicans*, 50%; *C. parapsilosis*, 32%; *C. glabrata*, 51%) (Table 2).

Prior systemic antifungal drug therapy was associated with 40% increased adjusted odds of *C. auris* fungemia; nevertheless, an effect ranging from a 20% decrease to a 2.5-fold increase is also consistent with our data (adjusted odds ratio [aOR] 1.4 [95% CI 0.8–2.3]). A central venous catheter in situ also independently increased the odds of *C. auris* infection 2-fold (aOR 1.8 [95% CI 1.05–3.01]). Admission to a private-sector facility increased the odds of *C. auris* candidemia 3-fold (aOR 2.7 [95% CI 1.5–4.7]). Older patients (aOR 1.01 [95% CI 1.01–1.03] for every year) with longer hospitalization before the first positive blood culture (aOR 1.01 [95% CI 1.01–1.02] for every day admitted) were more likely to have *C. auris* fungemia.

To understand whether inherent differences between healthcare sectors influenced risk factors, we stratified *C. auris* data by healthcare sector (Table 3). In the public sector, prior antifungal drug therapy (especially azole therapy) was associated with 2-fold increased odds of *C. auris* bloodstream infection (aOR 2.0 [95% CI 1.0–3.9]; p = 0.04) after adjustment for patient age, sex, length of hospital stay, previous hospitalization, and presence of a central venous catheter in situ.

**Table 3:** Demographic and clinical characteristics of patients with *Candida auris* candidemia, by healthcare sector, South Africa, 2016–2017*

Characteristic	Public-sector hospitals, n = 99	Private-sector hospitals, n = 695	p value†
Age, y, median (IQR)	27 (2–42)	58 (44–70)	<0.001
Sex			0.64
Men and boys	63/99 (64)	221/364 (61)	NA
Women and girls	36/99 (36)	143/364 (39)	NA
Length of hospital stay, d, median (IQR)	49 (30–72)	68 (40–140)	0.03
Length of stay to first positive blood culture, d, median (IQR)	26 (13–42)	35 (16–58)	0.21
Hospital admission in past 12 mo	37/62 (60)	40/42 (95)	<0.001
Intensive care unit admission	54/68 (79.4)	56/57 (98.3)	0.001
Mechanical ventilation	21/52 (40)	23/39 (59)	0.09
Central venous catheter in situ	40/54 (74)	29/38 (76)	1.0
Total parenteral nutrition	22/52 (42)	15/38 (39)	0.83
Systemic antimicrobial drug therapy <14 d before positive culture	36/52 (69)	41/42 (98)	<0.001
Systemic antifungal drug therapy <14 d before positive culture‡	14/53 (26)	16/42 (38)	0.27
Azole	12/14 (85.7)	4/16 (25)	0.001
Polyene/amphotericin B	4/14 (28.6)	3/16 (18.8)	0.68
Echinocandin	0/14 (0)	13/16 (81.3)	<0.001
Crude in-hospital mortality ratio	22/59 (37)	24/43 (56)	0.07

*Values are no. (%) except as indicated. Age data were available for 435 patients, and data on sex were available for 428 patients. For the rest of the variables, data were available for only a small proportion of patients from enhanced surveillance sites (total, N = 110; public sector, n = 67; private sector, n = 43). IQR, interquartile range; NA, not applicable. †Proportions were compared by using a χ^2^ or Fisher exact test; medians were compared by using the Wilcoxon rank-sum test. ‡Patients could have received >1 class of antifungal drug therapy.

## Discussion

In conducting this comprehensive national survey, we found that *C. auris* caused >10% of all cases of candidemia in South Africa and was the third most common *Candida* species. The incidence of *C. auris* candidemia was highest in private-sector hospitals in Gauteng Province. The crude in-hospital case-fatality ratio did not differ between *Candida* species. Prior systemic antifungal drug therapy was associated with increased adjusted odds of *C. auris* fungemia compared with candidemia caused by other species, and this effect was stronger in public-sector hospitals.

*C. auris* has rapidly emerged as a major cause of candidemia in South Africa, surpassing the number of cases caused by *C. glabrata*, *C. tropicalis*, and *C. krusei* over the past 7 years. A clear shift has occurred in the epidemiology observed from a previous national survey during 2009–2010 and a recent dramatic increase in the number of cases of *C. auris* invasive infection and colonization nationwide (*1*,*12*). We speculate that delayed clinician and laboratory awareness might have led to undetected transmission of the pathogen early in the epidemic (*16*).

The incidence of *C. auris* candidemia was highest in hospitals in Gauteng Province and is partly attributable to ongoing and recurrent clusters in these hospitals during the surveillance period. We speculate that the epidemic in South Africa might be centered in this area because of a combination of complex and interdependent healthcare system and behavioral factors, including a highly concentrated and mobile patient population; a large number of referrals and admission of patients with clinically complex cases to hospitals in the region; indiscriminate use of antimicrobial agents, including azoles and echinocandins; and suboptimal infection prevention and control practices. In addition, international travel to and from Gauteng Province might also play a role, as suggested by recent case reports and outbreaks in other continents caused by the South Africa clade of *C. auris* (*5*,*17*–*20*). In the United States, 90% of clinical cases of *C. auris* occurred in the New York metropolitan area, and most patients had lengthy hospitalizations in facilities that had capacity for highly skilled nursing and mechanical ventilation (*21*), suggesting that a large susceptible population of severely ill patients within a facility might provide a starting point for an outbreak that is then amplified by transmission. Individual hospital outbreaks seemed to overlap in Gauteng Province, suggesting that interfacility and intersectoral transmission of infections might have occurred; however, we have not yet established epidemiologic links among cases from different facilities. Whole-genome sequencing to establish molecular links is under way to more clearly characterize the epidemiology of *C. auris* candidemia in South Africa.

Prior systemic antifungal drug use was associated with *C. auris* candidemia, particularly in public-sector hospitals. This finding is consistent with data from similar studies and is probably related to selective pressure by azoles (*6*). Almost all tested *C. auris* isolates from South Africa are resistant to fluconazole (*2*) (T.G. Maphanga, NICD, pers. comm., 2018 Jul 27). Fluconazole is commonly used as a first-line treatment option, especially in public-sector hospitals, where access to echinocandin antifungal drugs is currently limited. The forthcoming 2019 guidelines for treatment of *C. auris* in South Africa recommend echinocandins as a first-line treatment for candidemia and amphotericin B deoxycholate if echinocandins are unavailable (*22*). In contrast to other *Candida* species, such as *C. parapsilosis*, for which a substantial proportion of infections occur among the neonatal population, *C. auris* occurs among older adults (*12*). In South Africa, an outbreak among 6 neonates in a neonatal unit has been documented (*23*), and several other small outbreaks have occurred (N.P. Govender, unpub. data). To date, no neonatal cases have been reported from the United States or Europe, although India, Colombia, and Venezuela have reported cases (*4*–*6*,*19*,*24*,*25*). Whether this phenomenon is attributable to inherent factors of the pathogen, environmental factors in neonatal units, or chance is still unclear. Nevertheless, we should be proactive to not let *C. auris* establish a foothold in neonatal units in developing countries as *C. parapsilosis* has done (*12*,*26*).

In the unique healthcare environment of South Africa, patients admitted to private-sector facilities were more likely to have *C. auris* candidemia. We hypothesize that this might be attributable to early undetected outbreaks in this sector, inherent differences in the patient populations admitted, or structural differences in the 2 healthcare sectors; more patients with *C. auris* candidemia at private-sector facilities were mechanically ventilated, had prior hospitalization, and had prior systemic antimicrobial drug therapy. Antimicrobial drug prescription behavior and differences in antimicrobial drug stewardship practices, including easier access to a broader range of antifungal drugs, might also play a role. Last, ongoing outbreaks at a few facilities might drive the higher case numbers in the private healthcare sector. The presence of a central venous catheter is a well-established risk factor for bloodstream infections (*27*). It is not surprising that central venous catheters were associated with *C. auris* candidemia because the pathogen has been shown to form biofilms and adhere to polymeric surfaces (*8*,*10*).

To address the continued transmission of *C. auris* in health facilities in South Africa, *C. auris* has been identified as a priority pathogen for surveillance to monitor emergence of antifungal drug resistance from all infection sites. We have also adapted published laboratory methods for rapid identification of *C. auris* colonization in the context of outbreak investigations (*28*). Local studies are also being planned to investigate the efficacy of novel antifungal agents (*29*).

This study had several limitations. We analyzed data for laboratory-confirmed candidemia only and did not include patients with other invasive *Candida* infections, culture-negative sepsis, or colonization, which might underestimate the extent of the problem in South Africa. However, 18%–22% of reported cases of *C. auris* infection in Europe and South Africa are bloodstream infections, and 58% of clinical isolates in the United States are from blood (*1*,*19*,*30*). In addition, 77% of cases of *C. auris* infection reported in the international literature are cases of candidemia; therefore, our study provides a plausible representation of the epidemiology of *C. auris*, albeit just the proverbial tip of the iceberg (*3*). The determination of incidence risk was based on data from a limited number of hospitals with admissions data available, mostly from the private sector. Therefore, we might have underestimated the incidence risk in the public sector. The reference laboratory confirmed the species identification of bloodstream isolates from only 45% of all detected cases of candidemia. Most cases without a reference laboratory species identification (70%) were from the private sector and had been detected retrospectively through audits. However, we believe that these national surveillance data still provide an accurate representation of the actual distribution of *C. auris* candidemia cases across sectors because most private laboratories used MALDI-TOF mass spectrometry methods to confirm *Candida* species identification. For cases at enhanced surveillance sites, we were limited to the availability of secondary data collected through an established surveillance program; we were unable to assess the duration of exposure to certain factors, such as parenteral nutrition and type of prior antimicrobial drug exposure. In addition, the linking of audit cases to reported cases was limited by demographic data available; therefore, we might have included duplicate cases in our analysis. Misclassification error might have occurred, given that a proportion of isolates did not have a species-level identification.

*C. auris* was the third most common cause of candidemia in South Africa and caused 14% of all cases during 2016–2017. Ongoing and recurrent micro-outbreaks might have driven the larger epidemic centered in Gauteng Province. Individual patient and healthcare risk factors should be considered when managing patients with suspected candidemia. The use of molecular epidemiology is needed to further characterize outbreaks in South Africa and better understand transmission dynamics of this emerging pathogen.
